# Effect of Freeze-Dried Porcine Platelet Lysate on Wound Healing in Rats

**DOI:** 10.3390/medicina61061098

**Published:** 2025-06-17

**Authors:** Winson Min-Teng Low, Yi-Ho Hsieh, Yi-Chieh Chu, Jui-Ting Hsiao, Yi-Ting Shu, Hung-Maan Lee, Ming-Fa Hsieh

**Affiliations:** 1 Department of Biomedical Engineering, Chung Yuan Christian University, No. 200, Zhongbei Rd., Zhongli Dist., Taoyuan City 320314, Taiwan; lowmit1988@hotmail.com; 2 Department of Orthopedics, Min-Sheng General Hospital, No. 168, Jingguo Rd., Taoyuan Dist., Taoyuan City 330056, Taiwan; dilantin11@gmail.com (Y.-H.H.); lala19930106@gmail.com (Y.-C.C.); bj22001@outlook.com (J.-T.H.); 3 Department of Materials and Textiles, Asia Eastern University of Science and Technology, No. 58, Sec. 2, Sihchuan Rd., Banciao District, New Taipei City 220303, Taiwan; yi_ting@cycu.org.tw; 4 Department of Orthopedics, Hualien Tzu Chi Hospital, Buddhist Tzu Chi Medical Foundation, No. 707, Zhongyang Rd., Sec. 3, Hualien City 970374, Taiwan; hungmaan01@gmail.com; 5 School of Medicine, Tzu Chi University, No. 701, Zhongyang Rd., Sec. 3, Hualien City 970374, Taiwan; 6 Department of Orthopedics, Taoyuan Armed Forces General Hospital, No. 168, Zhongxing Rd., Longtan Dist., Taoyuan City 325208, Taiwan

**Keywords:** wound healing, porcine platelet lysate, xenogeneic, in vivo study

## Abstract

*Background and Objectives:* Complications in wound healing present significant challenges in clinical settings. While platelet-rich plasma from human sources has been extensively used to aid wound recovery, allogeneic or xenogeneic platelet-derived products remain in the research phase. This study aimed to assess both the immunogenicity and therapeutic potential of xenogeneic porcine platelet lysate (pPL) in wound healing, using a rat model. *Materials and Methods:* Porcine platelet lysates with undetectable levels of antigens, including blood cells and complement factors, were engineered. Rat models simulating wound conditions were employed to investigate the effects of xenogeneic pPL on injured skin tissues. Histological assessments, including re-epithelialization, angiogenesis, and inflammatory cell response, were comprehensively conducted to evaluate the healing process. *Results:* The application of xenogeneic pPL on rat skin incisions significantly expedited the wound healing process. No rejection reaction was observed. Histological examinations of the xenogeneic pPL-treated wounds revealed enhanced re-epithelialization and angiogenesis compared to the wounds in control groups. *Conclusions:* These findings support the clinical promise of xenogeneic pPL as a feasible and effective agent for wound repair and tissue regeneration. This study suggests that its potential application in in vivo regeneration appears viable and promising.

## 1. Introduction

Skin constitutes 10% of the total body mass and is the largest organ of the body [1,2]. It possesses remarkable self-repair and renewal capabilities essential for defense and survival and functions as a vital barrier between the external and internal environments. Any disruption to the normal structure and function of the skin is termed a wound [3]. Healing of wounds involves a coordinated and dynamic process of tissue repair through the intricate interplay of growth factors, various cell types, chemokines, and cytokines [4,5]. Interruption in wound healing may result in developing non-healing wounds or excessive granulation tissue [6]. Wound healing is a complex biological process involving inflammation, cell proliferation, angiogenesis, and extracellular matrix remodeling [7]. Despite advances in wound care, chronic and non-healing wounds remain a major clinical challenge, particularly in patients with underlying conditions such as diabetes or vascular disease. Therefore, there is a continued need for effective, biocompatible therapies that can accelerate and enhance tissue regeneration.

Platelet-rich plasma (PRP) is applied widely across diverse medical domains. PRP amplifies the wound-healing mechanism in wounds and facilitates tissue regeneration. This is accomplished by supplying the necessary growth factors and cytokines required to activate cell proliferation and differentiation [8,9]. The advantage of autologous PRP is the avoidance of rejection issues [10]. Nevertheless, the clinical application of autologous PRP is not replete with challenges as it involves time-consuming procedures and potential variability in the concentration of growth factors [10]. Furthermore, the need for collecting substantial amounts of blood poses an additional health burden for patients [11]. Types of medical devices and isolation procedures also largely influence variations in platelet count enrichment, adding to the complexity of autologous PRP [12,13,14].

Due to the above challenges associated with autologous PRP, the development of allogeneic platelet-derived formulations has been widely explored. However, allogeneic PRP faces challenges due to the presence of residual antigens that may cause rejection reactions. Hence, platelet lysate (PL) emerges as a promising approach to eliminate these antigens. Platelet lysate disrupts the platelet membranes in PRP through physical methods [10]. Platelet fragments and large proteins are filtered out, followed by heating to remove complements, ensuring the elimination of potential antigens while extracting growth factors from platelets [10].

Platelet lysate emerges as a promising asset in the field of regenerative medicine. Platelet lysate offers high lot-to-lot consistency, a cost effective price structure, and can be produced in large quantities [15]. Platelet lysate is derived from platelet concentrate, and serves as a versatile repository of various growth factors and cytokines like platelet-derived growth factor (PDGF), transforming growth factor-beta (TGFβ), and vascular endothelial growth factor (VEGF) [16,17,18]. This array of growth factors is crucial for the proliferation, migration, and chemotaxis of various cell types essential to the process of wound healing [8,19,20]. Particularly, the external application of PL, in vitro or in vivo, demonstrates substantial effects on cell migration and proliferation [21].

Compared to allogeneic platelet lysate, xenogeneic platelet lysate is more easily accessible and more cost-effective while retaining their advantages. Xenogeneic and allogeneic platelet lysates share the same advantage of eliminating residual antigens that may trigger rejection reactions while retaining essential growth factors [10]. There is substantial clinical evidence supporting the use of autologous platelet lysate products, but further research is required to validate the efficacy of allogeneic and xenogeneic platelet lysate. To our knowledge, this is the first study to evaluate the wound healing effects of porcine platelet lysate in a rat excisional wound model, with a focus on both efficacy and potential immune response. This research holds the potential to advance future wound healing treatments and extend regenerative treatments for humans and other tissues using xenogeneic platelet lysate [11].

In this study, we developed a xenogeneic platelet lysate derived from porcine sources, taking advantage of the abundant availability of porcine blood from adult animals and optimized production procedures [15]. We formulated porcine platelet lysate (pPL), which is free of detectable antigens, such as blood cells and complement. The porcine platelet lysate was administered to full-thickness skin wounds in rats. The primary objective of this study was to evaluate the effect of freeze-dried porcine platelet lysate (PL) on the wound healing process in rats, including assessment of wound closure area and histological tissue regeneration. The secondary objectives included analyzing the biocompatibility of porcine platelet lysate to determine its potential as a safe and effective therapeutic biomaterial.

## 2. Materials and Methods

### 2.1. Study Objectives and Research Questions

This in vivo pilot study was designed to investigate the regenerative potential of freeze-dried porcine platelet lysate in full-thickness skin wounds in rats. The primary objectives were to evaluate wound closure and histological healing. The secondary objectives were to determine the biocompatibility of the PL treatment.

The key research questions included are as follows:

- Can freeze-dried porcine PL improve the rate and quality of wound healing?
- Is the treatment associated with enhanced collagen formation?
- Is porcine PL a biocompatible and well-tolerated material for skin application in rats?

### 2.2. Platelet Lysate Preparation

Porcine PL was prepared in accordance with the methodology outlined by Jonsdottir-Buch SM et al. [22], with minor modifications. In the current work, the sequential process involved centrifugation of porcine whole blood (500 RCF for 10 min), extraction of the supernatant, filtration to remove leukocytes (SAFETRAN Leukocyte Reduction Filter/Transfusion sets), and lysis of platelets through the freeze–thaw method [10]. Next, the solution was heated at 56 °C for 30 min to eliminate the complement, followed by filtration using a 0.22 µm filter (catalog number PC602-0050, GeneDireX™, TAQKEY Science, New Taipei City, Taiwan). The filtered PL was then lyophilized to improve stability and extend shelf life. The lysate was aliquoted into sterile, depyrogenated glass vials and pre-frozen at −80 °C for 24 h. Lyophilization was performed using a freeze-dryer (Christ Alpha 1-2 LDplus, Martin Christ, Gefriertrocknungsanlagen GmbH, Osterode am Harz, Germany) at −40 °C under a vacuum pressure of approximately 0.040 mbar for 48 h. The lyophilized powder was stored at 4 °C under sterile conditions.

A blood cell count was performed at the diagnostic department of Min-Sheng General Hospital; the results showed that the number of red blood cells, white blood cells, and platelets in the lysate was all 0. The concentration of growth factors in the lysate was determined using a TGF-β enzyme-linked immunosorbent assay (ELISA) kit. For this study, TGF-β was selected as the representative growth factor for detection. TGF-β concentration in samples was measured using the TGF-β DuoSet ELISA Kit (catalog number DY240-05, R&D Systems, Minneapolis, MN, USA) and R&D DuoSet Ancillary Reagent Kit 1 (catalog number DY007, R&D Systems, Minneapolis, MN, USA) following the recommended protocols. After TGF-β concentration in each batch of extracted pPL was determined, 0.05 mg of freeze-dried PL with a known content (25 ng) of TGF-β was measured and diluted with normal saline for use in animal experiments.

### 2.3. Animal Study

This study was designed as a pilot investigation, consistent with the ARRIVE 2.0 guidelines [23,24], to explore the potential effects of porcine platelet lysate (PL) on wound healing in vivo. A completed ARRIVE 2.0 guidelines checklist is provided as Supplementary File. A total of three rats were used, each receiving two wounds (one control and one experiment), employing a within-subject (paired) design to minimize inter-animal variability. Pilot studies are commonly conducted with small sample sizes to evaluate feasibility, optimize protocols, and detect biologically relevant trends before proceeding to larger-scale studies. While no formal power calculation was performed for this exploratory work, the sample size was informed by previous literature using similar models in preliminary wound healing experiments [25]. The results serve to justify and guide future studies with increased animal numbers and statistical power.

A total of three 6-week-old male Sprague-Dawley rats (weighing 250–300 g) from BioLASCO Taiwan Co., Ltd. were used in this pilot study. The animal experiments were performed according to the guidelines of ISO 10993-6:2016 edition [26] (IACUC Approval No. 23T10-13, approved on 28 July 2023, Master Laboratory Co., Ltd., Taipei City, Taiwan). They were quarantined and acclimatized for a week before the treatment. A veterinarian ensured the healthy status of the experimental animals before the treatment. Rats were housed individually (1 rat per cage) throughout the quarantine, acclimation, and experimental periods. All animals were maintained under standardized environmental conditions with a 12:12 h light-dark cycle, ambient temperature of 23 ± 3 °C, and humidity of 50 ± 20%. Lab Diet^®^ 5001 Rodent Diet (Lab Diet, PMI Nutrition International, LLC, ST.Louis, MO, USA) and reverse osmosis (RO) water were provided ad libitum.

**Figure 1:**
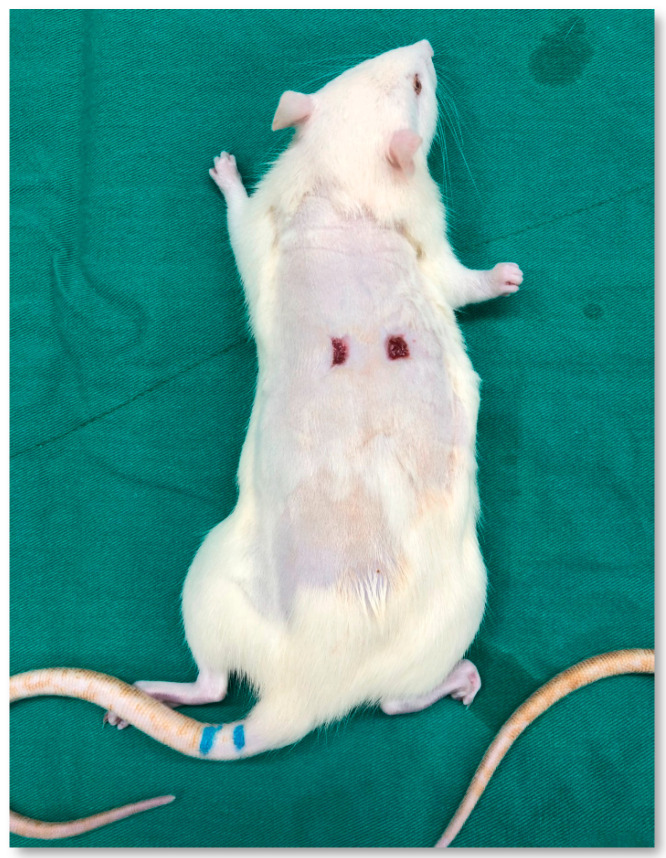
Rat with two wounds on its back before application of platelet lysate. The left wound serves as the control group, while the right wound serves as the experimental group.

After 1 week of acclimatization, experimental animals were anesthetized by intramuscular injection of Zoletil^®^ 100 (10 mg/kg) and xylazine (10 mg/kg), followed by maintenance with inhaled isoflurane. For wounds created by skin incision, the dorsal fur of the animals was first clipped in one direction using an electric shaver. Two full-thickness excisional wounds, each measuring 1.5 cm × 1.5 cm in diameter and approximately 0.5 cm in depth, were created on the dorsal skin of each rat using a scalpel and tissue scissors, as illustrated in Figure 1. To the right wound on the back of each rat, pPL was applied and covered with gauze. The following day, the gauze was removed, fresh pPL and new gauze were applied. This procedure was repeated for 14 days, while the left wound on the back of each rat was left untreated and served as the control group. This intra-animal paired design was selected to minimize biological variability and allow direct comparison of treatment effects within the same subject.

During the wound healing period, animals were maintained under the same controlled environmental conditions, including a temperature of 23 ± 3 °C, relative humidity of 50 ± 20%, and a 12:12 h light–dark cycle. Food and reverse osmosis (RO) water were provided ad libitum. In the experimental period, the wounds were observed for signs of redness, swelling, and discharge. The normalcy of physiological functions was assessed, including changes in diet and weight.

After 14 days of topical pPL application, the wounds were photographed, and tissue from the wounded area was obtained. Wound area percentages were recorded at days 0, 1, 4, 7, 10, and 14. The excised tissues were subjected to thorough histological assessments to evaluate the healing process, encompassing parameters such as re-epithelialization, angiogenesis, and inflammatory cell response. The tissues were stained with hematoxylin–eosin (H&E). The control and treated wound groups were compared in terms of re-epithelialization and the thickness of granulation tissue.

## 3. Results

Upon the application of pPL to the wound, there were no reactions or changes in diet or weight in these mice. Table 1 presents the body weight of each rat on days 0, 7, and 14. In our previous research [10], pPL obtained through our method did not induce rejection reactions in rabbit cartilage lesions.

**Figure 2:**
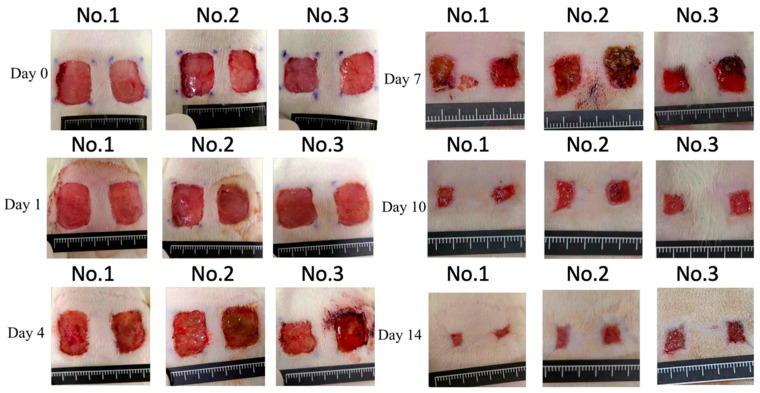
Wounds at days 0, 1, 4, 7, 10, and 14. The wounds on the left side represent the control group, while the wounds on the right side represent the experimental group.

Wound images of each group on days 0, 1, 4, 7, 10, and 14 are presented in Figure 2, while the corresponding percentage of the wound area is presented in Table 2. The wound area of the experimental and control groups did not differ significantly.

**Figure 3:**
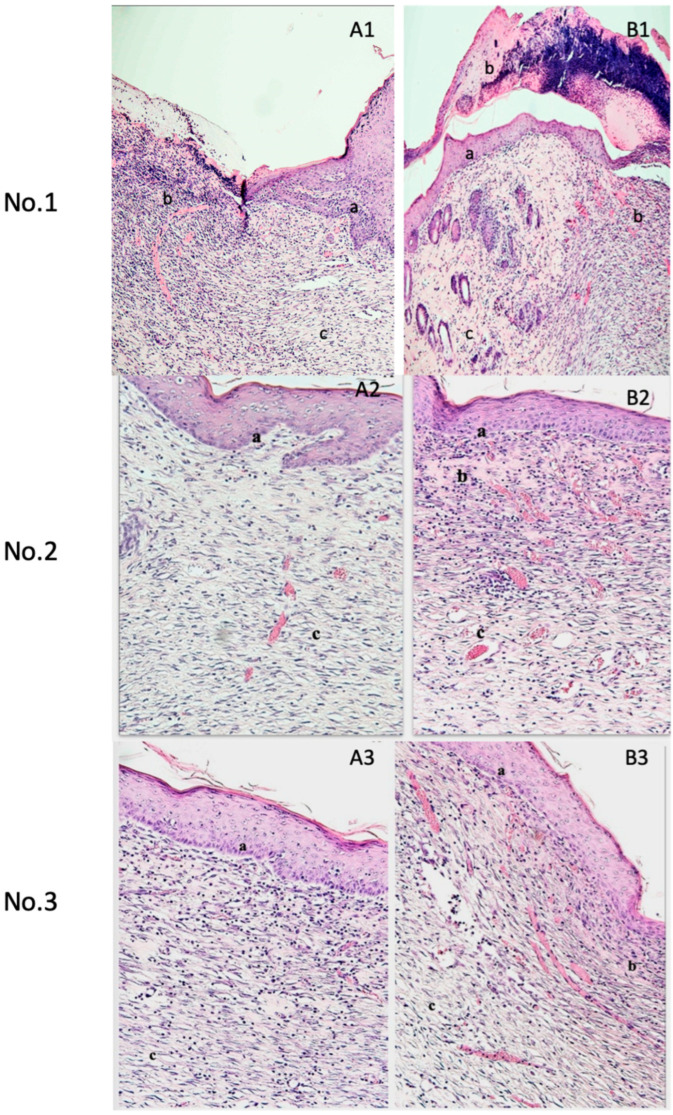
Left image **A** is hematoxylin and eosin staining of the control group; right image **B** is hematoxylin and eosin staining of the experimental group. The experimental group showed enhanced re-epithelialization, increased granulation tissue, and higher formation of capillaries in the wounds. (**a**) Epithelial tissue, (**b**) granulation tissue, (**c**) fibrous tissue.

Histological examination through H&E staining of day 14 samples demonstrated enhanced re-epithelialization and increased granulation tissue in the wounds treated with pPL. The pPL-treated wounds revealed an exclusive, newly formed epidermal layer. Furthermore, the qualitative assessment revealed enhanced capillary formation in the PL group compared to the control group. Figure 3 presents the histological examination of H&E-stained sections across all treatment groups.

## 4. Discussion

### 4.1. Perspectives for Clinical Practice

This study suggests that porcine platelet lysate (pPL) may accelerate wound healing by promoting re-epithelialization and granulation tissue formation. These results offer promising translational implications for clinical practice, particularly in the management of acute and chronic wounds. Wound healing is a complex, multidisciplinary process involving surgeons, dermatologists, and regenerative medicine specialists. The growth factors and cytokines in porcine platelet lysate could complement or replace conventional treatments, particularly for patients with impaired healing, such as those with diabetes or burns.

Platelet-derived products have gained interest for their regenerative abilities. Recent clinical and preclinical studies have demonstrated the efficacy of autologous regenerative strategies in severe burns and chronic wounds [27,28]. Although our study was conducted in a rat model, the observed biological effects of porcine platelet lysate provide a foundation for future translational research, including formulation development, dosing optimization, and safety assessment in human subjects.

### 4.2. Therapeutic Mechanisms and Histological Effects of Porcine Platelet Lysate

Different blood derivatives, such as cellular and molecular, play essential roles in wound healing. PRP or PL is the molecular component of blood derivatives and demonstrates therapeutic potency in wound healing. In the past, due to the lack of standardized sources and PL preparation methods, the therapeutic effect of PL was not significant (auto-, allo- or xenogeneous) [29]. Later, blood derivatives from donors were pooled to cancel the therapeutic variability [30]. Subsequently, the standard protocol for human platelet lysate was reported after decades of research [31]. Moreover, PL is regarded a better treatment option than PRP, with the advantages of PL over PRP being multifold. For example, the freeze and thaw cycles can be engineered to obtain a constant concentration of growth factors, and lyophilized PL can be stably stored for a certain time.

In this study, no rejection reactions were noted. Our method of PL preparation is designed so that most antigenic components, such as blood cells and proteins, are eliminated, as reported in previous studies [10]. Blood count and biochemical analysis of our PL preparation method confirmed the complete removal of blood cells, with only trace amounts of total protein and albumin. These findings highlight the efficacy of our approach in minimizing potential immunogenicity and the purity of the resulting PL used in the study.

Histological examination demonstrated faster healing of the pPL-treated wound. Platelet-derived products, including PRP, PL, and platelet gel, have found clinical applications to enhance wound healing [32]. These products are employed in the treatment of various skin conditions such as ulcers and diabetic wounds and as antibacterial agents [33,34]. Platelet derivatives provide a rich source of cytokines, growth factors (e.g., PDGF, TGF-β, VEGF), and chemokines that target both resident and recruited immune cells involved in wound healing [35,36]. Notably, the early recruitment and activation of macrophages and neutrophils is crucial for effective pathogen clearance, debris removal, and orchestration of the repair cascade [35]. By enhancing these early immune events, platelet lysate may help regulate the inflammatory phase and promote a more efficient transition to the proliferative and remodeling phases of healing [35]. Growth factors have been in use since the 1940s to enhance cutaneous wound healing [37]. They can be administered in various ways, including traditional topical or intralesional applications, specialized scaffolds, or gene therapy [37]. Animal and human trials have both documented successful clinical applications of PRP in the treatment of burn wounds [38], chronic skin ulcers [39,40], and full-thickness skin wounds [41].

Wound healing broadly occurs in four phases: hemostasis, inflammation, proliferation, and tissue remodeling [42]. In the hemostasis phase, platelets release alpha granules containing growth factors and cytokines [8], while immediately after wound injury, PDGF is released. PDGF acts as a chemotactic agent for fibroblasts, neutrophils, and monocytes. It promotes the production of extracellular matrix and collagen by fibroblasts and their transformation into myofibroblasts, causing granulation tissue formation and collagen matrix contraction. PDGF further stimulates macrophages to produce more growth factors, such as TGF-β, thereby contributing to the inflammatory phase of wound healing [43,44,45,46]. In the hemostasis phase, platelets also produce EGF, which is later also secreted by macrophages and fibroblasts. EGF serves as a chemotactic agent for keratinocytes and stimulates their proliferation, which is essential for re-epithelialization [44].

TGF-β exists in three isoforms, TGF-β1, TGF-β2, and TGF-β3. During wound healing, TGF-β1 is the most abundant [47]. TGF-β strongly promotes angiogenesis [43], regulates matrix deposition by fibroblasts and stimulates the synthesis of extracellular matrix components like hyaluronic acid, fibronectin, and collagen [47]. Platelets also release insulin-like growth factor (IGF) in the hemostasis phase. IGF attracts leukocytes and plays a regulatory function in fibroblast proliferation during the inflammatory and proliferative phases [47].

VEGF promotes angiogenesis, neo-vasculogenesis, and vascular permeabilization [43]. It promotes the migration of inflammatory cells and marrow-derived progenitor cells into the wound site and drives scar tissue deposition [48,49]. These growth factors position PL as a key player in activating in vivo regenerative processes.

Various techniques have been developed to enhance wound healing, targeting different stages of the repair process. Conventional approaches include the use of topical antibiotics, debridement, and moist dressings to prevent infection and support natural healing [50]. More advanced methods involve growth factor therapies, such as platelet-derived products, which supply cytokines and signaling molecules to stimulate cell proliferation, angiogenesis, and extracellular matrix formation [8,43,44]. Skin grafts and tissue-engineered constructs provide structural support for wound closure, especially in extensive or chronic wounds. Negative pressure wound therapy (NPWT) creates a controlled vacuum environment that promotes tissue granulation and fluid drainage [51]. Additionally, stem cell-based therapies and biomaterial scaffolds have emerged as promising regenerative strategies, offering cellular support and controlled release of bioactive factors [52]. Among these, platelet lysate-based treatments stand out for their autologous or xenogeneic origin, rich growth factor content, and low immunogenicity, making platelet lysate a versatile and scalable candidate in wound management.

Recent studies have debated the effectiveness of platelet-derived products [53,54,55,56]. The inconsistency in published results is likely due to the suboptimal quality of platelet-derived products resulting from inadequate and unstandardized procedures [36]. Substantial inter-individual variability in blood and platelet composition among patients also influence the effectiveness. Indeed, PL has emerged as a promising replacement for PRP in certain applications [57]. One key advantage of PL is the enhanced composition consistency between batches, leading to less variability and a more standardized product [58].

Several previous studies have explored the use of platelet lysate in promoting wound healing, primarily using human-derived and fresh preparations. For example, Yueh-Chen Chen et al. (2024) demonstrated that human platelet lysate exerts therapeutic effects on wound healing [59]. Similarly, Kameel Zuniga et al. (2022) [60] demonstrated that keratin hydrogels loaded with human platelet lysate exhibit wound-healing potential through prolonged delivery of regenerative factors. However, these studies did not address the feasibility of using xenogeneic sources such as porcine platelet lysate. Our study is the first to investigate the wound-healing effects of porcine platelet lysate in an in vivo rat wound model, assessing not only wound closure and collagen deposition but also the potential for immunological reaction. The use of porcine platelet lysate provides a more accessible and potentially scalable alternative to human products and enhances stability and shelf life.

In this study, PL-treated wounds displayed enhanced granulation tissue and capillary formation but no significant differences in wound area. One potential outcome of this observation is the impairment of epithelial cell proliferation during the removal of old gauze. In the experimentation period, pPL was applied to the wound on the back of each rat and covered with gauze. The following day, the gauze was removed, and fresh pPL and new gauze were applied. This procedure was repeated for 14 days. Consequently, epithelial cells may be potentially injured, and the wound bled when the old gauze was removed. Future animal experiments could aid in understanding and addressing this variable better and provide valuable insights. Although the macroscopic wound area did not significantly differ between groups, the potential advantage of porcine platelet lysate lies in its ability to enhance the biological quality of wound healing. Previous studies have shown that PL supports cell migration, angiogenesis, and extracellular matrix remodeling [7], which are not always immediately apparent through surface wound size alone. In our pilot study, histological findings suggest enhanced granulation tissue and capillary formation in the PL-treated wounds, supporting its future potential as a regenerative biologic.

### 4.3. Limitation

One primary limitation of the study is the relatively small sample size. A second limitation involves the daily gauze changes, which may have led to mechanical disruption of the wound bed, causing bleeding and potentially impairing epithelial cell proliferation, along with the potentially incomplete understanding of the long-term effects of xenogeneic PL. Additionally, the current study was limited to a 14-day observation period. While this timeframe captured the active phases of wound healing, extending the study to 21 days in future work would allow a more complete evaluation of wound healing. Another limitation is the absence of Masson’s Trichrome staining, which is widely used to specifically visualize collagen fibers and connective tissue remodeling. While H&E staining provided general histological insights, future studies will incorporate Masson’s Trichrome and other specialized stains to more comprehensively evaluate extracellular matrix composition and wound maturation. Nonetheless, our study supports the potential application of xenogeneic PL in clinical settings. Xenogeneic PL can be employed safely and effectively in wound healing after removing blood cells, complements, and large proteins from xenogeneic blood. Further research and clinical trials on humans may be warranted to validate and expand upon these preliminary findings.

## 5. Conclusions

Our study supports the use of xenogeneic platelet lysate in wound healing. Xenogeneic PL could safely and effectively promote tissue regeneration. The absence of rejection reactions, along with positive histological findings, including enhanced re-epithelialization and capillary formation, suggests its potential clinical application. While these results are promising, further research, including clinical trials, is needed to validate and facilitate the practical implementation of xenogeneic PL in wound healing treatments.

## Figures and Tables

**Table 1:** Individual body weight.

Animal No.	Gender	Body Weight (g)		
		Day 0	Day 7	Day 14
1	Male	275.3	306.3	313.2
2		272.1	295.9	313.9
3		271.2	309.4	321.3

**Table 2:** Percentage of wound size.

Group	Day 1	Day 4	Day 7	Day 10	Day 14
Control	100 ± 0%	81.2 ± 11.8%	36.6 ± 6.5%	13.8 ± 3.7%	10.7 ± 3.9%
Experiment	100 ± 0%	81.1 ± 6.9%	41.2 ± 6.7%	15.7 ± 3.8%	11.5 ± 4.3%

## Data Availability

The data presented in this study are available on request from the corresponding author.

## Supplementary Materials

The following supporting information can be downloaded at: https://www.mdpi.com/article/10.3390/medicina61061098/s1, Arrive Checklist.
